# An Assessment and Management of Plexiform Schwannoma of the Third Webspace in a Female: A Case Report

**DOI:** 10.7759/cureus.25305

**Published:** 2022-05-24

**Authors:** Abimbola O Ajibowo, Ojali R Unedu, Sheena Shiwlani, Olufemi S Ogunyemi, Toluwalope F Ejiyooye, Aadil Khan

**Affiliations:** 1 Internal Medicine, Lugansk Medical University, Lugansk, UKR; 2 Internal Medicine, University of Jos, Jos, NGA; 3 Pathology, Isra University, Karachi, PAK; 4 Pathology, QDx Pathology Services, Edison, USA; 5 Public Health, University of North Texas Health Science Center (UNTHSC), Fort Worth, USA; 6 Family Medicine, Brooke Army Medical Center, San Antonio, USA; 7 Internal Medicine, Lala Lajpat Rai (LLR) Hospital, Kanpur, IND

**Keywords:** plexiform, webspace, histopathological examination, morton's neuroma, schwannomas

## Abstract

Schwannoma is a rare benign soft tissue tumor that appears like a neuroma based on its specific location and clinical features. We report a case of a plexiform schwannoma in a middle-aged woman who had a painful bump located in the third webspace on the dorsum of her right foot for the last four years. Initially, the swelling was thought to be Morton's neuroma based on location and clinical feature findings. The mass was resected and was sent for histopathological examination, revealing a plexiform schwannoma, most likely developing from the cutaneous nerves on the dorsum of the foot. She reported improvement in her symptoms after complete and careful excision without any neurological deficit. Investigation of any subcutaneous foot swelling should be coupled with a histopathological examination for comprehensive management.

## Introduction

Plexiform schwannoma, also known as neurilemmomas, is a benign and uncommon presentation of peripheral nerve sheath growth. The tumor mainly originates from the slow proliferation of Schwann cells [1]. These nerve tumors are present in various locations, but the most common being the head, neck, or upper extremities [2]. This tumor mainly occurs in the third-fourth decade age group and is equally observed in both genders, and these tumors are typically less than 2 cm in size [3]. The predisposing factors for this condition are positive family history, history of trauma, and neurofibromatosis type 2 (NF-2) [4]. Peripheral schwannoma can have pain and paresthesia in the sensory distribution of the involved nerve; however, a large tumor can cause a motor deficit. Peripheral schwannoma is diagnosed using ultrasound and magnetic resonance imaging (MRI). Excision of the tumor and, at the same time, intactness of nerve function is highly monitored during operation. Herein, we underline a rare case of benign schwannoma of the dorsal cutaneous nerve located at the third webspace, which initially mimicked Morton's neuroma based on the clinical characteristics and location.

## Case presentation

**Figure 1:**
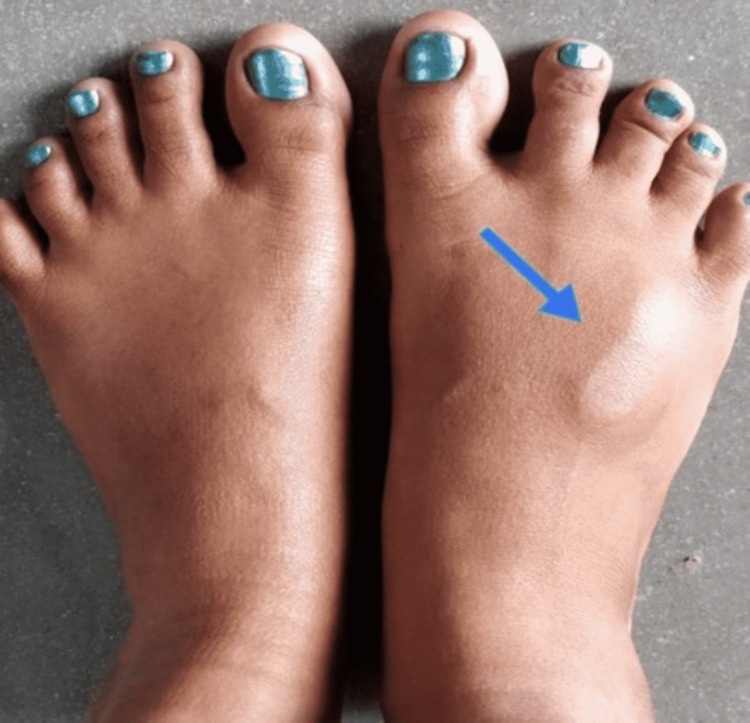
Focal swelling on the dorsum of the right foot.

**Figure 2:**
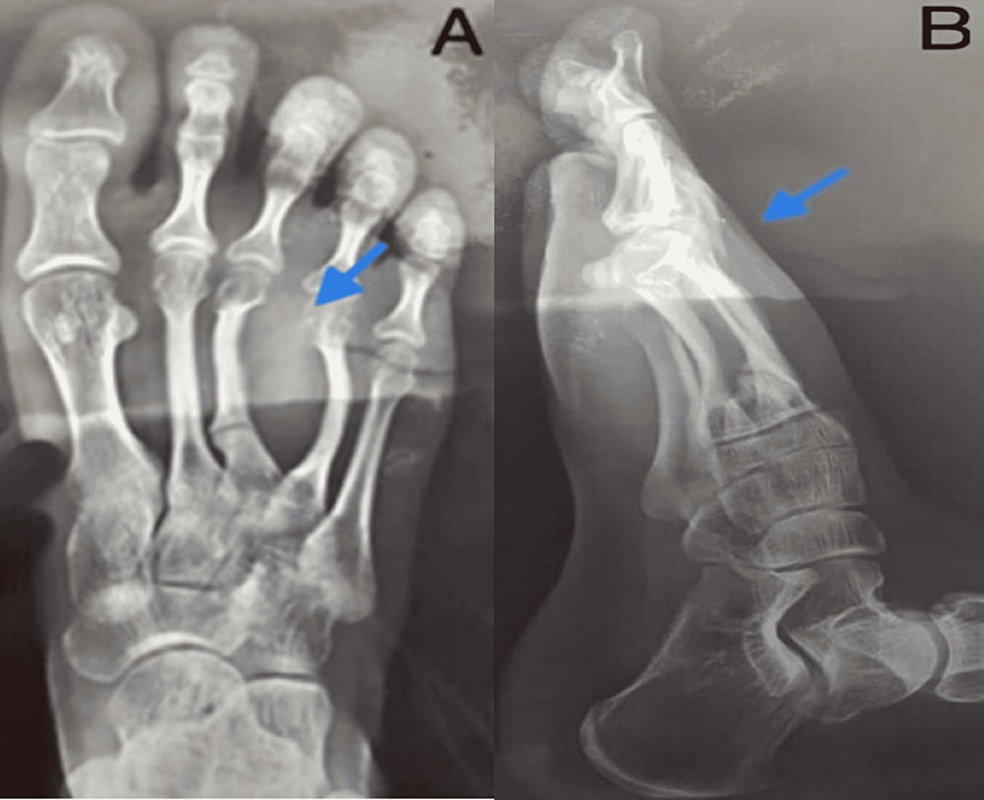
Plain right foot x-ray shows abnormal growth between the third and fourth digit in anteroposterior view (A) and lateral view (B).

A 37-year-old female without any past medical history presented with a swelling on the dorsum of her right foot for the past four years (Figure 1). The swelling was associated with pain, gradual onset, progressive, and worsened on walking. The physical evaluation of the right foot revealed swelling between the third and fourth digits; however, no muscle weakness and loss of sensation in the affected area were noted. A plain radiograph of the right foot showed abnormal growth in Figure 2.

Subsequently, an ultrasound of the dorsum of the right foot was done, showing a well-defined lobulated, homogenous hypoechoic lesion of size 4.7 × 3.5 cm is seen between the third and fourth digit of the right foot, along with posterior acoustic enhancement and intralesional flow noted, however, no cystic and calcification changes were present. Considering clinical lesion features, imaging, and location, a provisional diagnosis of a lesion of neural origin, possibly Morton's neuroma, was made. Her biochemical and hematological parameters were nonsignificant. After taking informed consent, the patient was managed operatively by excision of mass while maintaining continuity of the nerve fibers. This was performed under spinal anesthesia. She reported no neural deficit or any neural complication after excision. Post-operatively patients were managed by intravenous fluids, antibiotics, antacids, analgesics, and other symptomatic drugs. The regular aseptic dressing was done with the drain in situ. After two days, the patient was discharged with proper advice for a high-protein balanced diet, adequate bed rest, and regular follow-up.

**Figure 3:**
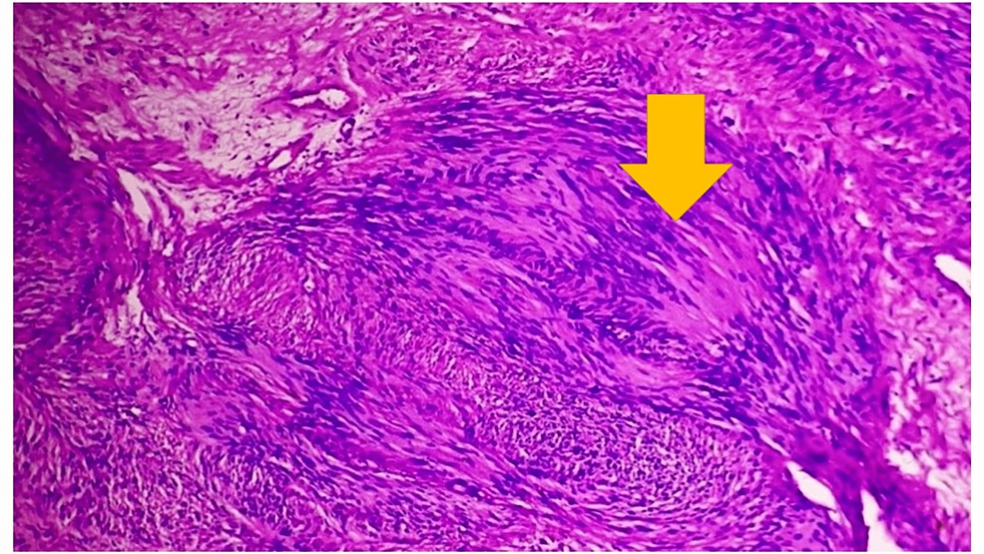
Histopathological examination shows spindle cells arranged in a parallel array with eosinophilic cytoplasm.

Her microscopic examination of the sections revealed tissue lined by unremarkable to sightly hyperplastic epidermis. The underlying stroma showed a partially well-encapsulated tumor composed of cellular areas alternating with paucicellular myxoid areas showing loosely dispersed spindle cells. The cellular areas showed fascicles of spindle cells arranged primarily on organoid whorls or lying parallel and occasionally also haphazardly. In the cellular areas, there were spindle cell nuclei arranged in a well-defined palisade manner around eosinophilic areas, suggesting the formation of verocay body structures. The spindle cells showed mild anisonucleosis, had an oval to elongated buckled or polypoidal nucleus with vesicular or occasionally slightly darkly stained chromatin, and abundant pale to deeply eosinophilic cytoplasm stretched out at both ends. A few thick and thin-walled blood vessels were also seen. No atypical mitosis is seen in Figure 3. Given the above findings of microscopic examination, the diagnosis of schwannoma of the right foot was made.

## Discussion

Schwannoma is a rare nerve sheath tumor that grows from Schwann cells. Schwann cell tumors are classified as schwannomas, malignant peripheral sheath tumors, and neurofibromas. Schwannomas make up 5% of all benign soft tissue neoplasms [5]. Schwannomas are also linked to many genetic diseases, including schwannomatosis, NF-2, and Carney complex type (CC-1) [6,7]. Clinically, this tumor is often observed as a small nodule that presents subcutaneously or intradermally; the highest diameter reported is mostly less than 2 cm.

The preoperative diagnosis of schwannoma is made by imaging modalities like ultrasonography and MRI. An ultrasound of a schwannoma shows it to be a homogenous, hypoechoic mass and may indicate its proximity to a nearby nerve. Occasionally, posterior acoustic enhancement and internal blood flow patterns can also be observed, as in our case [8,9]. An MRI of these benign tumors reveals hyperintense signals on T-2 weighted images and iso-tense signals on T-1 weighted images. However, it is not economically feasible and available in a resource-limited setting. The diagnosis is confirmed by histopathological examination in most clinical settings [10].

Schwannoma mainly presents as an asymptomatic solitary nodule with slow progression, which can be histologically categorized into plexiform, cellular, and melanotic variants [11]. The plexiform variant of schwannoma is a benign neoplasm that grows in a plexiform pattern; however, the chances for malignant variant are more likely in NF-2 and schwannomatosis [12]. In our case, the histopathological study confirmed the lesion as schwannoma, which was initially misdiagnosed as Morton's neuroma based on clinical and ultrasonographic profiles. In the histopathological study, tumors consist of hypercellular Antoni A and hypocellular Antoni B areas; Antoni A is characterized by nuclear palisading and verocay bodies. Contrary to schwannomas, plexiform schwannomas develop in a plexiform configuration, containing numerous interconnected fascicles and nodules in Antoni A areas. In addition, plexiform schwannomas have a protein marker in Schwann cells known as S100 protein [10-12].

Surgical excision is recommended for significant swelling and patients presenting with persistent symptoms. These tumors arising from the nerve sheath are characterized by the splaying of nerve fibers underlying them [6]. Therefore, careful surgical removal of the tumor is needed while preserving the continuity of cutaneous nerve fibers to prevent postoperative neurological deficits. Our patient responded well to the treatment, with an improved outcome.

## Conclusions

Plexiform schwannoma is a rare benign soft tissue neoplasm. It should be kept in the differential diagnosis while dealing with subcutaneous swelling of the foot. In our case, the clinical presentation of this tumor was misdiagnosed as Morton's neuroma, so for diagnostic accuracy, an excisional biopsy is a crucial step in confirming the diagnosis. Complete surgical excision is recommended to halt recurrence and for good functional outcomes.
